# Aqua(4-hydroxy­benzoato-κ*O*)(4-hydroxy­benzoato-κ^2^ *O*,*O*′)(1,10-phenanthroline-κ^2^ *N*,*N*′)zinc(II) monohydrate

**DOI:** 10.1107/S1600536808002249

**Published:** 2008-01-25

**Authors:** Li-Li Kong, Shan Gao, Li-Hua Huo, Seik Weng Ng

**Affiliations:** a School of Chemistry and Materials Science, Heilongjiang University, Harbin 150080, People’s Republic of China; b Department of Chemistry, University of Malaya, 50603 Kuala Lumpur, Malaysia

## Abstract

The Zn atom in the title compound, [Zn(C_7_H_5_O_3_)_2_(C_12_H_8_N_2_)(H_2_O)]·H_2_O, exists in a distorted *cis*-ZnN_2_O_4_ octa­hedral coordination geometry. One of the 4-hydroxy­benzoate anions chelates in a bidentate manner whereas the other is monodentate. The complex mol­ecules are linked through the uncoordinated water mol­ecules into a hydrogen-bonded sheet structure.

## Related literature

For related zinc bis­(4-hydroxy­benzoate) structures containing an *N*-heterocycle, see: Hökelek & Necefouglu (1996 ▶); Nadzhafov *et al.* (1981 ▶); Necefoğlu *et al.* (2002 ▶); Wang & Okabe (2005 ▶); Zheng *et al.* (2006 ▶).
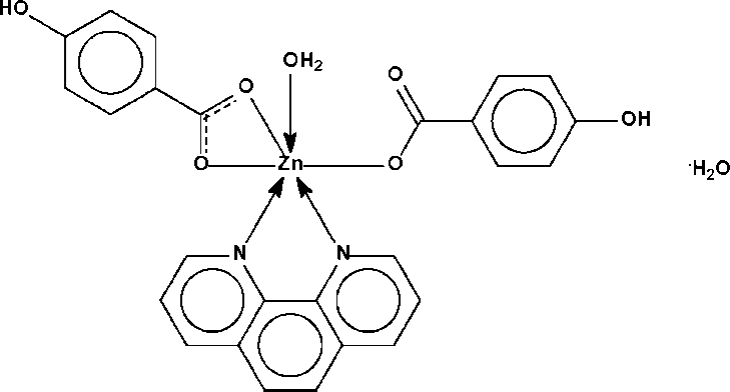


## Experimental

### 

#### Crystal data

[Zn(C_7_H_5_O_3_)_2_(C_12_H_8_N_2_)(H_2_O)]·H_2_O
*M* *_r_* = 555.83
Monoclinic, 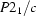

*a* = 11.1169 (5) Å
*b* = 19.738 (1) Å
*c* = 11.5503 (6) Å
β = 106.298 (1)°
*V* = 2432.5 (2) Å^3^
*Z* = 4
Mo *K*α radiation
μ = 1.06 mm^−1^
*T* = 295 (2) K
0.30 × 0.24 × 0.18 mm

#### Data collection

Rigaku R-AXIS RAPID diffractometer
Absorption correction: multi-scan (*ABSCOR*; Higashi, 1995 ▶) *T*_min_ = 0.674, *T*_max_ = 0.832
23098 measured reflections
5541 independent reflections
3924 reflections with *I* > 2σ(*I*)
*R*_int_ = 0.036

#### Refinement

*R*[*F* ^2^ > 2σ(*F* ^2^)] = 0.044
*wR*(*F* ^2^) = 0.131
*S* = 1.08
5541 reflections
336 parameters
H-atom parameters constrained
Δρ_max_ = 0.67 e Å^−3^
Δρ_min_ = −0.88 e Å^−3^

### 

Data collection: *RAPID-AUTO* (Rigaku, 1998 ▶); cell refinement: *RAPID-AUTO*; data reduction: *CrystalStructure* (Rigaku/MSC, 2002 ▶); program(s) used to solve structure: *SHELXS97* (Sheldrick, 2008 ▶); program(s) used to refine structure: *SHELXL97* (Sheldrick, 2008 ▶); molecular graphics: *X-SEED* (Barbour, 2001 ▶); software used to prepare material for publication: *publCIF* (Westrip, 2008 ▶).

## Supplementary Material

Crystal structure: contains datablocks global, I. DOI: 10.1107/S1600536808002249/hb2688sup1.cif

Structure factors: contains datablocks I. DOI: 10.1107/S1600536808002249/hb2688Isup2.hkl

Additional supplementary materials:  crystallographic information; 3D view; checkCIF report

## Figures and Tables

**Table 1:** Selected bond lengths (Å)

Zn1—O1	2.025 (2)
Zn1—O2	2.470 (2)
Zn1—O4	2.069 (2)
Zn1—O1*w*	2.143 (2)
Zn1—N1	2.075 (2)
Zn1—N2	2.155 (2)

**Table 2:** Hydrogen-bond geometry (Å, °)

*D*—H⋯*A*	*D*—H	H⋯*A*	*D*⋯*A*	*D*—H⋯*A*
O3—H3*o*⋯O2^i^	0.85	1.81	2.619 (3)	157
O6—H6*o*⋯O4^ii^	0.85	1.90	2.742 (3)	171
O1*w*—H1*w*1⋯O2*w*^iii^	0.85	2.01	2.819 (4)	158
O1*w*—H1*w*2⋯O5	0.85	1.84	2.599 (3)	148
O2*w*—H2*w*1⋯O5	0.85	2.04	2.801 (4)	148

Symmetry codes: (i) 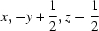; (ii) 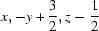; (iii) 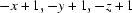.

**Fig. 1.**
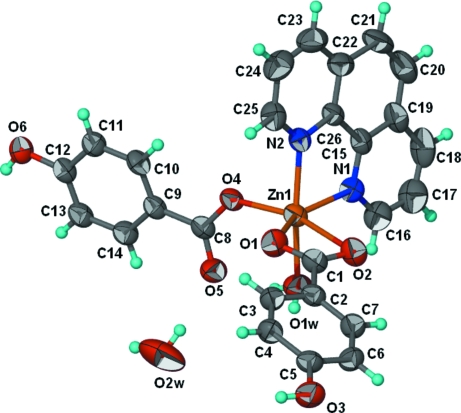
View of the molecular structure of (I) with displacement ellipsoids drawn at the 50% probability level (H atoms drawn as spheres of arbitrary radius).

## supplementary crystallographic information

### Comment

The title compound, (I), extends the range of adducts of zinc bis(4-hydroxybenzoate) with *N*-heterocycles: For the *N*,*N*-diethylnicotinamide adduct, see: Hökelek & Necefouglu (1996). For the pyridine adduct, see; Nadzhafov *et al.* (1981). For the nicotinamide adduct, see: Necefoğlu *et al.* (2002). For the bis(2-pyridyl)amine adduct, see: Wang & Okabe (2005). For the benzimidazole adduct, see: Zheng *et al.* (2006).

The Zn atom in (I) adopts a distorted *cis*-ZnN_2_O_4_ coordination geometry. One of the 4-hydroxybenzoate anions chelates in an anisobidentate manner whereas the other is unidentate (Table 1, Fig. 1).

In the crystal, the complex molecules are linked through the uncoordinated water molecules into a hydrogen-bonded sheet structure (Table 2).

### Experimental

Zinc diacetate dihydrate (1 mmol), 1,10-phenanthroline (2 mmol) and 4-hydroxybenzoic acid (2 mmol) were dissolved in aqueous ethanol Colourless blocks of (I) were isolated after several days.

### Refinement

The carbon-bound and hydroxyl H atoms were placed in calculated positions (C–H = 0.93, O–H = 0.85 Å) and refined as riding with *U*_iso_(H) 1.2–1.5*U*_eq_(C, O). The water H atoms were placed in chemically reasonable positions with O—H = 0.85Å on the basis of likely hydrogen bonding interactions and refined as riding with *U*_iso_(H) = 1.2*U*_eq_(O).

### Crystal data

**Table:**

[Zn(C_7_H_5_O_3_)_2_(C_12_H_8_N_2_)(H_2_O)]·H_2_O	*F*(000) = 1144
*M**_r_* = 555.83	*D*_x_ = 1.518 Mg m^−^^3^
Monoclinic, *P*2_1_/*c*	Mo *K*α radiation, λ = 0.71073 Å
Hall symbol: -P 2ybc	Cell parameters from 10359 reflections
*a* = 11.1169 (5) Å	θ = 3.1–27.5°
*b* = 19.738 (1) Å	µ = 1.06 mm^−^^1^
*c* = 11.5503 (6) Å	*T* = 295 K
β = 106.298 (1)°	Block, colorless
*V* = 2432.5 (2) Å^3^	0.30 × 0.24 × 0.18 mm
*Z* = 4	

### Data collection

**Table:**

Rigaku R-AXIS RAPID diffractometer	5541 independent reflections
Radiation source: fine-focus sealed tube	3924 reflections with *I* > 2σ(*I*)
graphite	*R*_int_ = 0.036
Detector resolution: 10.000 pixels mm^-1^	θ_max_ = 27.5°, θ_min_ = 3.1°
ω scans	*h* = −14→14
Absorption correction: multi-scan (*ABSCOR*; Higashi, 1995)	*k* = −25→25
*T*_min_ = 0.674, *T*_max_ = 0.832	*l* = −14→14
23098 measured reflections	

### Refinement

**Table:**

Refinement on *F*^2^	Primary atom site location: structure-invariant direct methods
Least-squares matrix: full	Secondary atom site location: difference Fourier map
*R*[*F*^2^ > 2σ(*F*^2^)] = 0.044	Hydrogen site location: inferred from neighbouring sites
*wR*(*F*^2^) = 0.131	H-atom parameters constrained
*S* = 1.08	*w* = 1/[σ^2^(*F*_o_^2^) + (0.0632*P*)^2^ + 0.8555*P*] where *P* = (*F*_o_^2^ + 2*F*_c_^2^)/3
5541 reflections	(Δ/σ)_max_ = 0.001
336 parameters	Δρ_max_ = 0.67 e Å^−^^3^
0 restraints	Δρ_min_ = −0.88 e Å^−^^3^

### Fractional atomic coordinates and isotropic or equivalent isotropic displacement parameters (Å^2^)

**Table:**

	*x*	*y*	*z*	*U*_iso_*/*U*_eq_	
Zn1	0.23086 (3)	0.517846 (16)	0.72570 (3)	0.05090 (13)	
O1	0.2083 (2)	0.43474 (11)	0.6190 (2)	0.0681 (6)	
O2	0.2863 (2)	0.40272 (11)	0.8070 (2)	0.0663 (6)	
O3	0.3803 (2)	0.13818 (12)	0.5298 (2)	0.0729 (6)	
H3o	0.3548	0.1357	0.4533	0.109*	
O4	0.19613 (18)	0.60020 (9)	0.61024 (17)	0.0507 (4)	
O5	0.3820 (2)	0.61864 (13)	0.5776 (2)	0.0725 (6)	
O6	0.03862 (19)	0.81782 (11)	0.19250 (19)	0.0612 (5)	
H6o	0.0924	0.8392	0.1669	0.092*	
O1w	0.4267 (2)	0.52299 (12)	0.7387 (2)	0.0749 (7)	
H1w1	0.4483	0.4874	0.7080	0.112*	
H1w2	0.4409	0.5576	0.7007	0.112*	
O2w	0.5117 (3)	0.5725 (2)	0.4171 (4)	0.1517 (16)	
H2w1	0.4623	0.5704	0.4614	0.182*	
H2w2	0.4914	0.6047	0.3664	0.182*	
N1	0.2575 (2)	0.55676 (12)	0.8980 (2)	0.0524 (6)	
N2	0.0408 (2)	0.52319 (11)	0.7369 (2)	0.0478 (5)	
C1	0.2581 (3)	0.38959 (15)	0.6961 (3)	0.0555 (7)	
C2	0.2870 (3)	0.32281 (14)	0.6520 (3)	0.0498 (6)	
C3	0.2367 (3)	0.30452 (15)	0.5320 (3)	0.0559 (7)	
H3	0.1830	0.3342	0.4795	0.067*	
C4	0.2653 (3)	0.24292 (16)	0.4895 (3)	0.0589 (7)	
H4	0.2291	0.2308	0.4095	0.071*	
C5	0.3479 (3)	0.19928 (15)	0.5664 (3)	0.0549 (7)	
C6	0.4005 (3)	0.21700 (15)	0.6863 (3)	0.0574 (7)	
H6	0.4563	0.1879	0.7381	0.069*	
C7	0.3693 (3)	0.27787 (15)	0.7279 (3)	0.0555 (7)	
H7	0.4039	0.2893	0.8085	0.067*	
C8	0.2667 (3)	0.62967 (14)	0.5549 (3)	0.0495 (6)	
C9	0.2074 (3)	0.67968 (14)	0.4592 (2)	0.0480 (6)	
C10	0.0776 (3)	0.68671 (15)	0.4180 (3)	0.0539 (7)	
H10	0.0269	0.6598	0.4508	0.065*	
C11	0.0234 (3)	0.73289 (16)	0.3295 (3)	0.0562 (7)	
H11	−0.0634	0.7370	0.3031	0.067*	
C12	0.0982 (3)	0.77342 (14)	0.2796 (2)	0.0509 (6)	
C13	0.2280 (3)	0.76739 (15)	0.3202 (3)	0.0562 (7)	
H13	0.2786	0.7945	0.2874	0.067*	
C14	0.2815 (3)	0.72096 (14)	0.4094 (3)	0.0543 (7)	
H14	0.3683	0.7172	0.4366	0.065*	
C15	0.1503 (3)	0.56991 (13)	0.9281 (2)	0.0501 (6)	
C16	0.3666 (3)	0.57100 (18)	0.9768 (3)	0.0701 (9)	
H16	0.4399	0.5617	0.9560	0.084*	
C17	0.3757 (4)	0.5985 (2)	1.0862 (4)	0.0874 (12)	
H17	0.4542	0.6070	1.1392	0.105*	
C18	0.2708 (5)	0.6137 (2)	1.1192 (3)	0.0868 (12)	
H18	0.2774	0.6333	1.1940	0.104*	
C19	0.1501 (4)	0.59957 (16)	1.0383 (3)	0.0669 (9)	
C20	0.0339 (5)	0.61090 (19)	1.0621 (4)	0.0853 (12)	
H20	0.0331	0.6306	1.1350	0.102*	
C21	−0.0760 (4)	0.5941 (2)	0.9824 (4)	0.0843 (12)	
H21	−0.1505	0.6015	1.0022	0.101*	
C22	−0.0799 (3)	0.56479 (16)	0.8669 (3)	0.0640 (8)	
C23	−0.1891 (4)	0.5470 (2)	0.7791 (4)	0.0847 (12)	
H23	−0.2668	0.5547	0.7923	0.102*	
C24	−0.1823 (4)	0.5185 (2)	0.6742 (4)	0.0856 (12)	
H24	−0.2551	0.5069	0.6150	0.103*	
C25	−0.0643 (3)	0.50667 (18)	0.6557 (3)	0.0654 (8)	
H25	−0.0604	0.4866	0.5840	0.078*	
C26	0.0332 (3)	0.55205 (13)	0.8411 (2)	0.0472 (6)	

### Atomic displacement parameters (Å^2^)

**Table:**

	*U*^11^	*U*^22^	*U*^33^	*U*^12^	*U*^13^	*U*^23^
Zn1	0.0592 (2)	0.0422 (2)	0.0576 (2)	0.00058 (14)	0.02675 (17)	0.00114 (14)
O1	0.0915 (16)	0.0451 (11)	0.0708 (14)	0.0159 (11)	0.0279 (12)	0.0032 (10)
O2	0.0923 (16)	0.0487 (12)	0.0629 (13)	0.0041 (11)	0.0302 (12)	−0.0055 (10)
O3	0.0831 (16)	0.0597 (13)	0.0764 (14)	0.0212 (12)	0.0235 (13)	−0.0112 (12)
O4	0.0592 (11)	0.0410 (10)	0.0603 (11)	0.0033 (8)	0.0306 (9)	0.0069 (8)
O5	0.0613 (13)	0.0814 (16)	0.0829 (15)	0.0100 (12)	0.0336 (12)	0.0261 (13)
O6	0.0682 (13)	0.0559 (12)	0.0626 (12)	−0.0031 (10)	0.0232 (10)	0.0126 (10)
O1w	0.0624 (14)	0.0749 (16)	0.0959 (18)	0.0095 (11)	0.0359 (13)	0.0233 (13)
O2w	0.140 (3)	0.187 (4)	0.162 (3)	−0.004 (3)	0.099 (3)	−0.052 (3)
N1	0.0549 (14)	0.0441 (13)	0.0575 (13)	−0.0065 (10)	0.0145 (12)	−0.0008 (11)
N2	0.0518 (13)	0.0453 (12)	0.0484 (12)	−0.0067 (10)	0.0174 (11)	0.0025 (10)
C1	0.0639 (17)	0.0443 (15)	0.0639 (18)	−0.0001 (13)	0.0271 (15)	0.0002 (14)
C2	0.0550 (15)	0.0416 (14)	0.0586 (16)	0.0015 (12)	0.0257 (13)	0.0001 (12)
C3	0.0608 (17)	0.0522 (17)	0.0568 (16)	0.0093 (14)	0.0200 (14)	0.0030 (13)
C4	0.0648 (18)	0.0591 (18)	0.0544 (16)	0.0071 (15)	0.0193 (15)	−0.0058 (14)
C5	0.0582 (16)	0.0458 (15)	0.0656 (18)	0.0065 (13)	0.0254 (14)	−0.0047 (14)
C6	0.0605 (17)	0.0492 (16)	0.0620 (17)	0.0121 (13)	0.0162 (14)	0.0017 (14)
C7	0.0614 (17)	0.0499 (16)	0.0568 (16)	0.0013 (13)	0.0193 (14)	−0.0018 (13)
C8	0.0573 (16)	0.0418 (14)	0.0552 (15)	−0.0020 (12)	0.0254 (14)	−0.0023 (12)
C9	0.0580 (16)	0.0409 (14)	0.0505 (14)	−0.0050 (12)	0.0244 (13)	−0.0025 (12)
C10	0.0591 (17)	0.0531 (16)	0.0557 (16)	−0.0110 (13)	0.0263 (14)	0.0028 (13)
C11	0.0554 (16)	0.0565 (17)	0.0588 (17)	−0.0053 (13)	0.0197 (14)	0.0047 (14)
C12	0.0654 (17)	0.0408 (14)	0.0511 (15)	−0.0042 (12)	0.0241 (14)	−0.0017 (12)
C13	0.0648 (18)	0.0468 (15)	0.0663 (18)	−0.0070 (13)	0.0336 (15)	0.0051 (14)
C14	0.0562 (16)	0.0483 (16)	0.0630 (17)	−0.0055 (13)	0.0242 (14)	0.0016 (13)
C15	0.0711 (18)	0.0350 (13)	0.0475 (14)	−0.0020 (12)	0.0220 (14)	0.0025 (11)
C16	0.068 (2)	0.065 (2)	0.071 (2)	−0.0107 (17)	0.0100 (17)	−0.0038 (17)
C17	0.089 (3)	0.088 (3)	0.077 (2)	−0.018 (2)	0.009 (2)	−0.005 (2)
C18	0.136 (4)	0.065 (2)	0.0498 (18)	−0.021 (2)	0.011 (2)	−0.0128 (16)
C19	0.105 (3)	0.0459 (17)	0.0577 (18)	0.0020 (17)	0.0366 (19)	−0.0015 (14)
C20	0.138 (4)	0.063 (2)	0.071 (2)	0.011 (2)	0.055 (3)	−0.0033 (18)
C21	0.105 (3)	0.068 (2)	0.108 (3)	0.026 (2)	0.076 (3)	0.017 (2)
C22	0.0641 (19)	0.0555 (18)	0.082 (2)	0.0096 (15)	0.0358 (18)	0.0179 (16)
C23	0.062 (2)	0.085 (3)	0.115 (3)	0.0119 (19)	0.039 (2)	0.032 (3)
C24	0.056 (2)	0.092 (3)	0.099 (3)	−0.0126 (19)	0.005 (2)	0.020 (2)
C25	0.069 (2)	0.066 (2)	0.0607 (18)	−0.0145 (16)	0.0163 (16)	0.0010 (15)
C26	0.0586 (16)	0.0359 (13)	0.0534 (15)	0.0016 (11)	0.0259 (13)	0.0065 (11)

### Geometric parameters (Å, °)

**Table:**

Zn1—O1	2.025 (2)	C7—H7	0.9300
Zn1—O2	2.470 (2)	C8—C9	1.490 (4)
Zn1—O4	2.069 (2)	C9—C14	1.392 (4)
Zn1—O1w	2.143 (2)	C9—C10	1.394 (4)
Zn1—N1	2.075 (2)	C10—C11	1.376 (4)
Zn1—N2	2.155 (2)	C10—H10	0.9300
O1—C1	1.272 (4)	C11—C12	1.391 (4)
O2—C1	1.257 (4)	C11—H11	0.9300
O3—C5	1.359 (3)	C12—C13	1.392 (4)
O3—H3o	0.8501	C13—C14	1.383 (4)
O4—C8	1.282 (3)	C13—H13	0.9300
O5—C8	1.253 (3)	C14—H14	0.9300
O6—C12	1.358 (3)	C15—C19	1.402 (4)
O6—H6o	0.8501	C15—C26	1.447 (4)
O1w—H1w1	0.8500	C16—C17	1.353 (6)
O1w—H1w2	0.8501	C16—H16	0.9300
O2w—H2w1	0.8499	C17—C18	1.358 (6)
O2w—H2w2	0.8500	C17—H17	0.9300
N1—C16	1.326 (4)	C18—C19	1.431 (5)
N1—C15	1.357 (4)	C18—H18	0.9300
N2—C25	1.318 (4)	C19—C20	1.412 (6)
N2—C26	1.356 (3)	C20—C21	1.349 (6)
C1—C2	1.481 (4)	C20—H20	0.9300
C2—C3	1.388 (4)	C21—C22	1.443 (5)
C2—C7	1.394 (4)	C21—H21	0.9300
C3—C4	1.381 (4)	C22—C23	1.391 (5)
C3—H3	0.9300	C22—C26	1.394 (4)
C4—C5	1.384 (4)	C23—C24	1.357 (6)
C4—H4	0.9300	C23—H23	0.9300
C5—C6	1.389 (4)	C24—C25	1.406 (6)
C6—C7	1.374 (4)	C24—H24	0.9300
C6—H6	0.9300	C25—H25	0.9300
			
O1—Zn1—O4	105.98 (8)	O4—C8—C9	117.7 (2)
O1—Zn1—N1	147.52 (9)	C14—C9—C10	118.4 (3)
O4—Zn1—N1	106.14 (8)	C14—C9—C8	120.3 (3)
O1—Zn1—O1w	91.75 (10)	C10—C9—C8	121.3 (2)
O4—Zn1—O1w	90.57 (8)	C11—C10—C9	121.1 (3)
N1—Zn1—O1w	92.66 (10)	C11—C10—H10	119.5
O1—Zn1—N2	96.91 (9)	C9—C10—H10	119.5
O4—Zn1—N2	89.51 (8)	C10—C11—C12	120.0 (3)
N1—Zn1—N2	78.65 (9)	C10—C11—H11	120.0
O1w—Zn1—N2	170.97 (10)	C12—C11—H11	120.0
O1—Zn1—O2	57.39 (8)	O6—C12—C11	117.0 (3)
O4—Zn1—O2	161.68 (7)	O6—C12—C13	123.3 (3)
N1—Zn1—O2	91.30 (8)	C11—C12—C13	119.7 (3)
O1w—Zn1—O2	82.91 (8)	C14—C13—C12	119.7 (3)
N2—Zn1—O2	99.59 (8)	C14—C13—H13	120.1
C1—O1—Zn1	100.74 (19)	C12—C13—H13	120.1
C1—O2—Zn1	80.68 (17)	C13—C14—C9	121.1 (3)
C5—O3—H3o	109.5	C13—C14—H14	119.5
C8—O4—Zn1	130.40 (18)	C9—C14—H14	119.5
C12—O6—H6o	109.5	N1—C15—C19	122.5 (3)
Zn1—O1w—H1w1	109.4	N1—C15—C26	117.3 (2)
Zn1—O1w—H1w2	109.5	C19—C15—C26	120.1 (3)
H1w1—O1w—H1w2	109.5	N1—C16—C17	122.7 (4)
H2w1—O2w—H2w2	111.0	N1—C16—H16	118.7
C16—N1—C15	118.9 (3)	C17—C16—H16	118.7
C16—N1—Zn1	126.4 (2)	C16—C17—C18	120.4 (4)
C15—N1—Zn1	114.65 (18)	C16—C17—H17	119.8
C25—N2—C26	118.2 (3)	C18—C17—H17	119.8
C25—N2—Zn1	129.3 (2)	C17—C18—C19	119.6 (3)
C26—N2—Zn1	112.33 (18)	C17—C18—H18	120.2
O2—C1—O1	120.3 (3)	C19—C18—H18	120.2
O2—C1—C2	121.2 (3)	C15—C19—C20	118.5 (3)
O1—C1—C2	118.5 (3)	C15—C19—C18	115.8 (3)
O2—C1—Zn1	70.63 (16)	C20—C19—C18	125.7 (3)
O1—C1—Zn1	50.35 (14)	C21—C20—C19	122.1 (3)
C2—C1—Zn1	164.2 (2)	C21—C20—H20	118.9
C3—C2—C7	118.2 (3)	C19—C20—H20	118.9
C3—C2—C1	120.6 (3)	C20—C21—C22	121.0 (3)
C7—C2—C1	121.2 (3)	C20—C21—H21	119.5
C4—C3—C2	121.0 (3)	C22—C21—H21	119.5
C4—C3—H3	119.5	C23—C22—C26	117.0 (3)
C2—C3—H3	119.5	C23—C22—C21	124.7 (3)
C3—C4—C5	119.8 (3)	C26—C22—C21	118.3 (3)
C3—C4—H4	120.1	C24—C23—C22	120.0 (3)
C5—C4—H4	120.1	C24—C23—H23	120.0
O3—C5—C4	122.6 (3)	C22—C23—H23	120.0
O3—C5—C6	117.3 (3)	C23—C24—C25	119.5 (4)
C4—C5—C6	120.0 (3)	C23—C24—H24	120.2
C7—C6—C5	119.5 (3)	C25—C24—H24	120.2
C7—C6—H6	120.3	N2—C25—C24	122.0 (4)
C5—C6—H6	120.3	N2—C25—H25	119.0
C6—C7—C2	121.5 (3)	C24—C25—H25	119.0
C6—C7—H7	119.3	N2—C26—C22	123.4 (3)
C2—C7—H7	119.3	N2—C26—C15	116.8 (2)
O5—C8—O4	123.4 (3)	C22—C26—C15	119.8 (3)
O5—C8—C9	118.9 (2)		
			
O4—Zn1—O1—C1	166.06 (18)	C1—C2—C7—C6	−177.4 (3)
N1—Zn1—O1—C1	−22.8 (3)	Zn1—O4—C8—O5	−11.7 (4)
O1w—Zn1—O1—C1	75.0 (2)	Zn1—O4—C8—C9	168.48 (17)
N2—Zn1—O1—C1	−102.5 (2)	O5—C8—C9—C14	−9.9 (4)
O2—Zn1—O1—C1	−5.47 (18)	O4—C8—C9—C14	169.9 (3)
O1—Zn1—O2—C1	5.51 (18)	O5—C8—C9—C10	170.5 (3)
O4—Zn1—O2—C1	−21.3 (3)	O4—C8—C9—C10	−9.7 (4)
N1—Zn1—O2—C1	176.32 (19)	C14—C9—C10—C11	0.5 (4)
O1w—Zn1—O2—C1	−91.16 (19)	C8—C9—C10—C11	−179.9 (3)
N2—Zn1—O2—C1	97.61 (19)	C9—C10—C11—C12	0.1 (5)
O1—Zn1—O4—C8	−80.1 (2)	C10—C11—C12—O6	179.9 (3)
N1—Zn1—O4—C8	104.8 (2)	C10—C11—C12—C13	−0.5 (4)
O1w—Zn1—O4—C8	11.9 (2)	O6—C12—C13—C14	179.9 (3)
N2—Zn1—O4—C8	−177.1 (2)	C11—C12—C13—C14	0.3 (4)
O2—Zn1—O4—C8	−56.9 (4)	C12—C13—C14—C9	0.3 (5)
C1—Zn1—O4—C8	−70.8 (3)	C10—C9—C14—C13	−0.7 (4)
O1—Zn1—N1—C16	92.9 (3)	C8—C9—C14—C13	179.7 (3)
O4—Zn1—N1—C16	−95.9 (3)	C16—N1—C15—C19	1.6 (4)
O1w—Zn1—N1—C16	−4.6 (3)	Zn1—N1—C15—C19	−176.6 (2)
N2—Zn1—N1—C16	177.9 (3)	C16—N1—C15—C26	−178.4 (3)
O2—Zn1—N1—C16	78.4 (3)	Zn1—N1—C15—C26	3.4 (3)
C1—Zn1—N1—C16	80.4 (3)	C15—N1—C16—C17	−0.3 (5)
O1—Zn1—N1—C15	−89.0 (3)	Zn1—N1—C16—C17	177.7 (3)
O4—Zn1—N1—C15	82.14 (19)	N1—C16—C17—C18	−1.0 (6)
O1w—Zn1—N1—C15	173.51 (19)	C16—C17—C18—C19	1.0 (6)
N2—Zn1—N1—C15	−4.01 (18)	N1—C15—C19—C20	−179.6 (3)
O2—Zn1—N1—C15	−103.54 (19)	C26—C15—C19—C20	0.5 (4)
C1—Zn1—N1—C15	−101.5 (2)	N1—C15—C19—C18	−1.5 (4)
O1—Zn1—N2—C25	−33.1 (3)	C26—C15—C19—C18	178.5 (3)
O4—Zn1—N2—C25	73.0 (3)	C17—C18—C19—C15	0.2 (5)
N1—Zn1—N2—C25	179.5 (3)	C17—C18—C19—C20	178.1 (4)
O2—Zn1—N2—C25	−91.1 (3)	C15—C19—C20—C21	0.1 (5)
C1—Zn1—N2—C25	−62.0 (3)	C18—C19—C20—C21	−177.8 (4)
O1—Zn1—N2—C26	151.55 (17)	C19—C20—C21—C22	−1.4 (6)
O4—Zn1—N2—C26	−102.41 (18)	C20—C21—C22—C23	−178.9 (4)
N1—Zn1—N2—C26	4.16 (17)	C20—C21—C22—C26	2.2 (5)
O2—Zn1—N2—C26	93.56 (17)	C26—C22—C23—C24	0.2 (5)
C1—Zn1—N2—C26	122.68 (18)	C21—C22—C23—C24	−178.7 (4)
Zn1—O2—C1—O1	−8.6 (3)	C22—C23—C24—C25	0.5 (6)
Zn1—O2—C1—C2	168.4 (3)	C26—N2—C25—C24	0.4 (5)
Zn1—O1—C1—O2	10.5 (3)	Zn1—N2—C25—C24	−174.8 (2)
Zn1—O1—C1—C2	−166.6 (2)	C23—C24—C25—N2	−0.8 (6)
O2—C1—C2—C3	168.8 (3)	C25—N2—C26—C22	0.3 (4)
O1—C1—C2—C3	−14.2 (4)	Zn1—N2—C26—C22	176.3 (2)
O2—C1—C2—C7	−14.0 (4)	C25—N2—C26—C15	−179.7 (3)
O1—C1—C2—C7	163.1 (3)	Zn1—N2—C26—C15	−3.7 (3)
C7—C2—C3—C4	1.3 (4)	C23—C22—C26—N2	−0.6 (4)
C1—C2—C3—C4	178.7 (3)	C21—C22—C26—N2	178.3 (3)
C2—C3—C4—C5	−1.8 (5)	C23—C22—C26—C15	179.4 (3)
C3—C4—C5—O3	−179.6 (3)	C21—C22—C26—C15	−1.7 (4)
C3—C4—C5—C6	1.0 (5)	N1—C15—C26—N2	0.4 (4)
O3—C5—C6—C7	−179.1 (3)	C19—C15—C26—N2	−179.6 (2)
C4—C5—C6—C7	0.3 (5)	N1—C15—C26—C22	−179.6 (2)
C5—C6—C7—C2	−0.8 (5)	C19—C15—C26—C22	0.4 (4)
C3—C2—C7—C6	0.0 (4)		

### Hydrogen-bond geometry (Å, °)

**Table:**

*D*—H···*A*	*D*—H	H···*A*	*D*···*A*	*D*—H···*A*
O3—H3o···O2^i^	0.85	1.81	2.619 (3)	157
O6—H6o···O4^ii^	0.85	1.90	2.742 (3)	171
O1w—H1w1···O2w^iii^	0.85	2.01	2.819 (4)	158
O1w—H1w2···O5	0.85	1.84	2.599 (3)	148
O2w—H2w1···O5	0.85	2.04	2.801 (4)	148

Symmetry codes: (i) *x*, −*y*+1/2, *z*−1/2; (ii) *x*, −*y*+3/2, *z*−1/2; (iii) −*x*+1, −*y*+1, −*z*+1.
